# A 12-Week Cycling Training Regimen Improves Upper Limb Functions in People With Parkinson’s Disease

**DOI:** 10.3389/fnhum.2018.00351

**Published:** 2018-09-11

**Authors:** Alexandra Nadeau, Ovidiu Lungu, Arnaud Boré, Réjean Plamondon, Catherine Duchesne, Marie-Ève Robillard, Florian Bobeuf, Anne-Louise Lafontaine, Freja Gheysen, Louis Bherer, Julien Doyon

**Affiliations:** ^1^Centre de Recherche de l’Institut Universitaire de Gériatrie de Montréal, Montréal, QC, Canada; ^2^Functional Neuroimaging Unit, Montréal, QC, Canada; ^3^Department of Psychology, Université de Montréal, Montréal, QC, Canada; ^4^Department of Psychiatry, Université de Montréal, Montréal, QC, Canada; ^5^Centre for Research in Aging, Donald Berman Maimonides Geriatric Centre, Montréal, QC, Canada; ^6^Department of Electrical Engineering, École Polytechnique, Montréal, QC, Canada; ^7^McGill Movement Disorder Clinic, McGill University Health Centre, Montréal, QC, Canada; ^8^Department of Movement and Sport Sciences, Ghent University, Ghent, Belgium; ^9^Department of Medicine, Université de Montréal, Montréal, QC, Canada; ^10^Montréal Heart Institute, Montréal, QC, Canada

**Keywords:** Parkinson’s disease, exercise, upper limb function, aerobic, stationary bicycle

## Abstract

**Background:** It has been proposed that physical exercise can help improve upper limb functions in Parkinson’s disease (PD) patients; yet evidence for this hypothesis is limited.

**Objective:** To assess the effects of aerobic exercise training (AET) on general upper limb functions in sedentary people with PD and healthy adults (HA).

**Methods:** Two groups, 19 PD patients (Hoehn & Yahr ≤ 2) and 20 HA, matched on age and sedentary level, followed a 3-month stationary bicycle AET regimen. We used the kinematic theory framework to characterize and quantify the different motor control commands involved in performing simple upper-limb movements as drawing lines. Repeated measures ANCOVA models were used to assess the effect of AET in each group, as well as the difference between groups following the training regimen.

**Results:** At baseline, PD individuals had a larger antagonist response, a longer elapsed time between the visual stimulus and the end of the movement, and a longer time of displacement of the stylus than the HA. Following the 12-week AET, PD participants showed significant decreases of the agonist and antagonist commands, as well as the antagonist response spread. A significant group ^∗^ session interaction effect was observed for the agonist command and the response spread of the antagonist command, suggesting a significant change for these two parameters only in PD patients following the AET. Among the differences observed at baseline, only the difference for the time of movement remained after AET.

**Conclusion:** A 3-month AET has a significant positive impact on the capacity to draw lines in a more efficiency way, in PD patients, indicating an improvement in the upper limb motor function.

## Introduction

Parkinson’s disease (PD) is a neurodegenerative condition characterized by cardinal motor symptoms, such as tremor, rigidity, and bradykinesia (Goetz et al., 2008). These symptoms impact the movement and function of the upper limbs during everyday activities such as writing, self-care, and fine object manipulation. Past studies have also shown that PD patients present difficulties in force control, as well as in coordinating and controlling multiple tasks (Alberts et al., 1998). For example, they are impaired in modulating muscles activity, as reflected by an antagonist activation occurring earlier than normal, hence overlapping with actions from the agonist muscle (Pfann et al., 2001). Such disease-related functional alterations may thus explain the difficulties observed in fine motor skills and general upper movements in PD, hence leading to restrictions in autonomy and quality of life.

Parkinson’s disease is usually treated using medication (levodopa, dopamine agonists) and surgical intervention (deep brain stimulation). While these treatments are very effective upon initiation, their effectiveness diminishes over time and a range of side effects emerges. Physical exercise has been proposed as an adjuvant therapy and a complementary approach that could improve both motor and non-motor symptoms in PD (Goodwin et al., 2008; Speelman et al., 2011). Among the motor benefits, a few studies have suggested that exercise could be a good alternative intervention to improve upper limb function (Ridgel et al., 2009, 2012; Muller and Muhlack, 2010; Alberts et al., 2011). However, it is imperative to understand the mechanisms underlying its therapeutic impacts. In addition, the present study intends to investigate the association between upper limb function and other domains such as executive functioning and motor sequence learning (MSL) known to be impaired in PD patients and sensitive physical exercise.

To date, several studies have aimed to better understand the role of exercise on neurophysiological mechanisms regulating upper limb functions in PD population (Ridgel et al., 2009; Alberts et al., 2011; David et al., 2016). In Alberts et al. (2011), the Opening Container Task was used in PD participants before and after an 8-week forced exercise (FE) intervention that used a lower limb tandem cycling apparatus (Alberts et al., 2011). In comparison to a voluntary exercise (VE) group, the FE group showed an improvement in grip-load coupling and an increased rate of grip force production. The authors concluded that such training modality could be efficient in improving global motor functioning in people with PD. However, the specific mechanisms underlying such improvements are still unknown. In another study, David et al. (2016) showed that not only a 24-month of progressive resistance exercise did result in faster elbow movement velocity in PD participants, but also that such exercising program led to a normalized magnitude of agonist burst and an increased antagonist muscle activity, as measured with electromyography (EMG) (David et al., 2016).

The efficacy of exercise in improving upper limb functions in PD cannot be properly assessed unless we use appropriate and specific measurements of this function. To date, several tests have been developed to assess fine upper limb functioning. For instance, tasks requiring object manipulation, such as coin flipping or spinning, changing a combination lock, transferring small objects from point A to point B (Stewart et al., 2009), simple tapping tasks, or the Purdue pegboard test, have all been used to measure motoric functions in PD patients (Alberts et al., 2011). Even tasks simulating real-life activities, such as opening a container, have been tested (Alberts et al., 2011) and shown to be sensitive to coordination and motor control problems seen in PD patients, hence being a good indicator of the global upper limb function in this clinical population. Yet, these motor tasks focus mostly on speed of execution, and few of them actually assess the underlying neurophysiological mechanisms mediating motor functioning in this population. An ideal test would allow the characterization of the temporal activation pattern of muscles during a task involving the upper limb (David et al., 2016), such as EMG. However, this technique is not user-friendly in clinical settings as it requires EMG expertise and significant time to install electrodes on the different muscle groups required to carry out the array of motor tasks needed to measure motoric functions in PD.

In this paper, we sought to test whether an aerobic exercise training (AET) regimen could bring similar improvements on upper limb motor control using another movement velocity task, a line drawing task, and whether the effects of AET on the neurophysiological level could be inferred using the kinematic theory of human movements. This theory offers an alternative way to indirectly characterize and quantify the different motor control commands involved in performing simple upper-limb movements (Plamondon, 1995a,b, 1998; Plamondon and Alimi, 1997; Feng and Plamondon, 2003; Plamondon et al., 2003; Djioua and Plamondon, 2008). According to this model, the very large number of coupled neural and muscular cells constituting a given neuromuscular network generate an impulse response that converge toward a lognormal profile. The central nervous system (CNS) then takes advantage of this emerging behavior in order to control the velocity of an end effector in simple and complex tasks.

**FIGURE 1:**
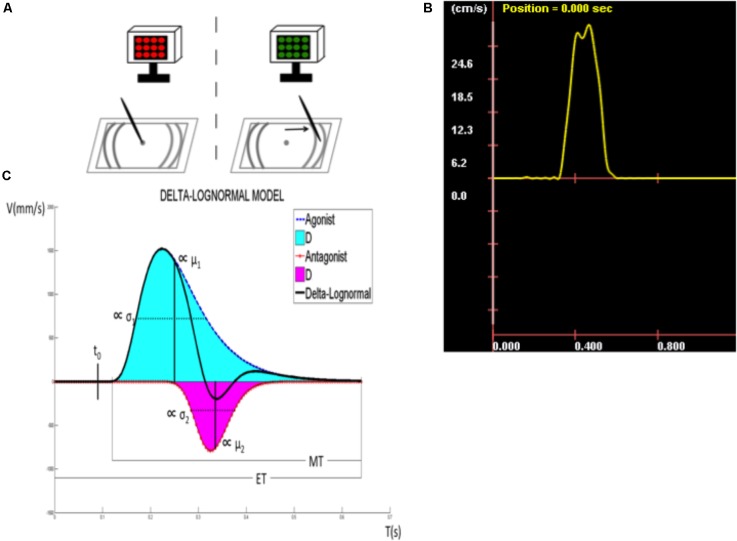
Description of the target directed fast simple RT task. **(A)** Tablet display and target zone. **(B)** Speed curve obtained for each line drawn. **(C)** Kinematic parameters (*t*_0_, time occurrence of the input commands to the neuromuscular system; *D*_1_, agonist component; μ_1_, time delay of *D*_1_; σ_1_, response spread of *D*_1_; *D*_2_, antagonist component; μ_2_, time delay of *D*_2_; σ_2_, response spread of *D*_2_; ET, time between visual stimulus and the immobilization of the stylus; RT, reaction time; MT, movement time).

**Figure 1C** illustrates, for example, how the Kinematic Theory describes a rapid pointing movement. Such a movement requires the activation of an agonist and an antagonist neuromuscular systems. Each of these systems is thought to produce a lognormal velocity profile, an asymetric bellshaped curve (solid line, agonist and dotted line, antagonist in **Figure 1C**) and the resulting velocity is the subtraction of these two curves, as depicted in **Figure 1B**, which is refered to as a delta-lognormal curve. Each delta-lognormal equation is described by seven parameters: *t*_0_, the time occurrence of the two input commands activating the pair of neuromuscular systems; *D*_1_ and *D*_2_, the agonist and antagonist commands; μ_1_ and μ_2_, the time delay of the agonist and antagonist systems (on a logarithmic scale); and σ_1_ and σ_2_, the time response of the agonist and antagonist systems (on a logarithmic scale). In other word, *t*_0_, *D*_1_, and *D*_2_ describe the central action plan and μ_1_, μ_2_, σ_1_, and σ_2_ the timing properties of the peripheral synergy reacting to it. Thus, according to the kinematic theory, the experimental delta-lognormal velocity profile (**Figure 1B**) can be used to reconstruct the given movement with its corresponding agonist and antagonist components, the seven parameters estimated during the reconstruction process allowing researchers to indirectly infer the properties of the central controller and the agonist and antagonist peripheral systems involved in such a movement.

The lognormality of the asymmetric bellshaped velocity profile has been validated in many comparative studies and under numerous experimental conditions (Plamondon et al., 1993; Djioua and Plamondon, 2008; Woch et al., 2011; O’Reilly et al., 2013). Moreover, it has even been mathematically demonstrated that the lognormal profile was the optimal output that a perfectly controlled neuromuscular system could produce (Djioua and Plamondon, 2010), and the basic hypotheses of this model have also been supported using electroencephalography (EEG) (O’Reilly et al., 2013) and EMG (Plamondon et al., 2013a) experiments, which have confirmed its physiological plausibility. Indeed it has been shown, using EEG, that a specific motor event-related potential (ERP) was happening at *t*_0_, the time occurrence of the neuromuscular commands, as predicted by the theory (O’Reilly et al., 2013). Additionally, the proportionality of the cumulative time delays between different muscles involved in a given movement have also been observed, as expected, from EMG measurements. Over the years, exploiting lognormal functions of the synergistic action of neuromuscular networks in numerous pointing tasks has proven to be a reliable way to describe the velocity profile of simple human movements (Plamondon et al., 1993; Plamondon, 1995a,b, 1998; Plamondon and Alimi, 1997; Djioua and Plamondon, 2008). In doing so, the parameters extracted in the signal reconstruction provided a global evaluation, albeit indirect, of a subject fine motricity, in terms of a central representation of his action plans and the timing properties of the peripheral processes reacting to it. In other words, without any EMG or EEG data capturing devices, the kinematic theory provides a consistent and non-invasive estimation of the global motor control behavior of a subject.

In response to the knowledge gap described above, the main objective of the current study was to assess the effects of AET on general upper limb functions in sedentary people with PD and healthy adults (HA). Although the exercise program aimed especially the lower limbs, we relied on the global effect of aerobic training to drive changes in upper limb function. In order to infer the CNS mechanisms underlying AET-related changes in upper-limb functions, we used a fast simple reaction time task (FSRTT) based upon the delta-lognormal model of the kinematic theory (O’Reilly and Plamondon, 2011; Plamondon et al., 2013a; O’Reilly et al., 2014). Indeed, in a straight line drawing task to a target, the delta-lognormal model is the simplest one to use. It can reproduce a given velocity profile by subtracting two lognormal curves, one representing the agonist activity toward this target and the other, the antagonist breaking at this target. We assumed that the kinematic theory (Plamondon, 1995a,b), with its delta-lognormal model, involving both agonist and antagonist activations during a simple line drawing task, would offer an efficient framework not only to assess the effect of exercise on upper limb function in PD patients, but also to simultaneously inform us, indirectly, on the mechanisms underlying its therapeutic impact. For example, if the AET had an impact on either *t*_0_, *D*_1_, and *D*_2_, this would mean that the training affected the central motor controller, whereas if AET had an impact on the μ_1_, μ_2_, σ_1_, and σ_2_, this would indicate that the peripheral system would be involved. Moreover, the present methodology allowed us to explore the agonist and antagonist systems separately. The data presented here are part of a larger research program that investigated the effects of this type of exercise training on several outcome measures such as cardiorespiratory capacities, executive functions, and MSL capacity (measured behaviorally and with functional imaging); the results of which have been presented elsewhere (Duchesne et al., 2015, 2016). Given that other types of outcome measures were collected before and after the intervention, the second objective of the current study was to investigate the possibility of correlations between exercise-related changes in upper limb function and other metrics related to motor symptoms, cardiovascular capacity, executive functions, and MSL capacity. We hypothesized that: (1) there would be differences at baseline between PD and their healthy counterparts regarding the kinematic properties of their agonist and antagonist neuromuscular systems, (2) these differences would diminish as a result of training, an effect driven specifically by changes in the PD group, who were expected to improve the kinematic parameters of their movements, (3) these improvements in upper limb movements would correlate with exercise-related changes in motor symptoms, cardiovascular capacity, executive functions, and MSL capacity.

## Materials and Methods

### Participants

Eighteen PD patients and 20 HA subjects, between 40 and 80 years of age, took part in the study. They were right-handed, sedentary [score of 5 or lower on the Jackson’s Questionnaire (Jackson et al., 1990)], neurologically intact [i.e., score of 24 or more on the Mini Mental State Evaluation (Folstein et al., 1975)], or the Montreal Cognitive Assessment (Marinus et al., 2011; Nasreddine et al., 2005). HA participants were matched with PD patients at the group level with respect to sex distribution, age, number of years of education, as well as cognitive and fitness levels. Exclusion criteria included other neurological disorders, comorbidities likely to affect gait, smoking, or heart diseases, and participation to <75% of the AET sessions during the study. PD patients had to be classified as stage 1 or 2 according to Hoehn and Yahr’s scale (1967) based upon evaluation of a certified neurologist (A-LL), and had to score below 35 on motor functions assessed with the third section of the United PD Rating Scale (UPDRS III) (Goetz et al., 2008). The target of 75% or more participation rate in the fitness training program had to be achieved by all participants to be retained in the analysis. This study was carried out in accordance with the recommendations of the research ethics committee’s guidelines of the Research Center of the “Institut Universitaire de Gériatrie de Montréal,” which approved the protocol. A written and informed consent was obtained from participants prior to their inclusion in this study.

### Exercise Intervention Protocol

Prior to engaging in the training regimen, all participants were cleared by a medical doctor, who analyzed the electrocardiogram (ECG) at rest and ruled out any cardiac anomalies that could put participants at risk during exercising. At the same time, all participants completed a graded exercise test with the stationary bicycle to obtain their peak oxygen uptake (VO_2_peak) (ACMS, 2006). The result at this test was used for personalized exercise prescription. The duration and frequency of AET was of 12 weeks, three times per week. Duration of the exercise sessions started at 20 min and 60% of intensity, and was then increased by steps of 5 min and 5% of intensity every week, until participants reached 40 min of training at 80% intensity. To reach a high-intensity level, bike speed was maintained at 60 revolutions per minute (RPM). As such, to achieve the desired bike resistance power and adjust intensity level (if needed), the work intensity was based on power output (Watt), controlling for subject’s heart rate. In addition, rate of perceived exertion (Borg scale) (Borg, 1982) was assessed during each training session. Even if some studies showed good results using FE (Ridgel et al., 2009, 2012, 2015; Alberts et al., 2011, 2016; Beall et al., 2013) to improve upper limb functions, we chose to use VE instead, because from a clinical and practical perspective, FE devices are not easily accessible to the general public, and because we wanted to test an easily accessible type of workout for this population. Trained kinesiologists supervised all training sessions.

### Assessments

Participants were evaluated on a set of outcome measures before the intervention (at baseline), and immediately after completion of the 3-month exercise program (post-intervention).

#### Main Outcome

Kinematic properties of the upper limb movement were assessed with a target-directed FSRTT using the kinematic theory (Plamondon et al., 1993, 2003; Plamondon, 1995a,b, 1998). This task employs an electronic drawing board (a graphic tablet), an electronic pen (stylus), and an electronic display to present visual stimuli. The tablet displays a dot in the center (starting position) and target zones on either side (**Figure 1A**). On each trial, participants are required to draw straight lines on the graphic tablet by executing simple arm movements in response to a visual stimulus. **Figure 1A** depicts the task and its phases: (1) A LED screen alternating from red to black indicates to the participants that the system is ready for acquisition. At this moment, the participant is asked to position the tip of the stylus on the starting position. (2) Once the stylus hits the digitizer, the LED screen stops blinking, turns black, and a green screen appears after a random delay, signaling the subject to start drawing a straight line toward the target zone as fast as possible. This delay is exponentially distributed, the parameters of the corresponding flat hazard distribution have been chosen such that the delay is between 0 and 10s. Thus, regardless of the duration the subject has waited for the stimulus, the probability that it will be emitted during the next millisecond is always the same (Luce, 1986). During line drawing, and once in the target zone, the pen has to be in contact with the tablet. (3) Once the stylus is in the target zone, the participants are required to keep it immobile and in contact with the tablet for a 3–5 s to allow for a better delimitation of the movement. After completing a trial, participants are asked to raise the stylus and wait for the screen to start alternating from black to red again signaling the onset of a new trial. Participants are informed that neither the precision, nor the direction of the movement are important, only the speed of execution. A speed curve is then obtained for each line drawn (**Figure 1B**). Trials in which participants did not reach the target zone or needed two segments to reach it were not counted and analyzed. Thirty successful trials, or a maximum of 40 trials, were required of all participants, whichever criterion was reached first. The mean of each kinematic parameter was calculated from successful trials and used as dependent variables (see the section “Extraction of the kinematic parameters of the movements”).

#### Secondary Outcomes

As secondary outcomes for the current study, we included the patient’s motor symptoms evaluation, assessed with the UPDRS (Goetz et al., 2008), sub-divided in scores for rigidity, tremor, motor symptoms for the right upper limb (including the following items: tremor at rest, postural tremor, rigidity of arm, finger taps, hand movements, rapid alternating movements of hands) in addition to a total score for this section of the questionnaire. Participants’ cardiovascular fitness level (VO_2_peak) was evaluated using a recumbent bike, either by a submaximal aerobic test (11 HA, 5 PD) or by a medically supervised maximal oxygen uptake test (9 HA, 14 PD). Mood was also evaluated using the Beck Depression Inventory (Beck et al., 1961) and the Beck Anxiety Inventory (Beck et al., 1988). The Stroop Test (naming, reading, interference) (Stroop, 1935) and the Trail Making Test (TMT A and B) (Sánchez-Cubillo et al., 2009) were used to evaluate inhibition and cognitive flexibility, respectively, two components of executive neuropsychological functions. In addition, participants’ MSL capacity was evaluated behaviorally using an implicit serial reaction time (RT) task performed during functional MRI acquisition. For more details regarding those evaluations, please refer to our previous published work (Duchesne et al., 2015, 2016).

### Extraction of the Kinematic Parameters of the Movements

Participants’ trials were used to extract several kinematic parameters of the movement based on the kinematic model (**Figure 1C**). Reconstruction of the velocity profile using two lognormal models was used to reveal the agonist and antagonist components of the profile. The upper curve (solid line) represents a measure of the agonist activity, which corresponds to the velocity of the pen tip toward its target. By contrast, the lower curve (dotted line) depicts the antagonist activity, which has a direct opposite contribution and is mainly used to break the motion, although it can also be useful in stabilizing the movement and in increasing its precision. The obtained velocity profile obeys the following delta-lognormal law (Plamondon et al., 1993, 2003; Plamondon, 1995a,b, 1998):

$$\upsilon (t)=D_{1}\Lambda_{1}(t;t_{0},\mu_{1},\sigma_{1}^{2})-D_{2}\Lambda_{2}(t;t_{0},\mu_{2},\sigma_{2}^{2}),$$

where

$$\Lambda (t;t_{0},\mu_{1},\sigma_{1}^{2})=\frac{1}{\sigma_{\text{i}}\sqrt{2\pi }(t-t_{0})}\exp-\left(\frac{{\left(\ln(t-t_{0})-{\text{μ}}_{i}\right)}^{2}}{2\sigma_{\text{i}}^{2}}\right),$$

and where *t*_0_ represents the time occurrence of the simultaneous input commands *D*_1_ and *D*_2_ to the neuromuscular system. The time between the occurrence of the stimulus (*t* = 0) and *t*_0_ is, in fact, the period needed for the perception of the stimulus and the command preparation. The delay between the stimulus onset (*t* = 0) and the beginning of the movement (beginning of the velocity increase) corresponds to the classical RT. The time between *t*_0_ and the RT corresponds to the command propagation time. In the upper curve, the area under the curve corresponds exactly to the agonist response (*D*_1_), while the μ_1_ and the σ_1_ represent, respectively, the time delay and the response spread of the agonist activation on a logarithmic scale. The equivalent is presented with the lower curve for the antagonist response (*D*_2_, μ_2_, σ_2_). In other words, *t*_0_, *D*_1_, and *D*_2_ reflect command processes, often referred to the action plan, in terms of amplitude and time occurrence, while μ_1_, σ_1_, μ_2_, and σ_2_ reflect the distributed timing properties of the system. The elapsed time (ET) corresponds to the delay between the moment where the visual stimulus is sent until the immobilization of the stylus on the digitizer, while the moment where the movement is started (beginning of the curve), until the immobilization of the stylus on the tablet is considered as the movement time (MT). Signal-to-noise ratio (SNR) between the original and the reconstructed velocity profile can be considered like a cue of the reconstruction’s quality. As suggested in O’Reilly et al. (2013), a SNR of 20 dB minimum is required to use the trial in the analysis, without that, the reconstruction was considered of too low quality. Also, trials with a negative *t*_0_ were rejected from analyzes. This situation may occur when the protocol is not respected in a given trial and a movement is anticipated, that is the commands are initiated before the onset of the stimulus. This might also seldom happen when the parameter extraction algorithm fails. Indeed, *t*_0_ is computed from the curve fitting process using a seven parameter optimization algorithm that minimizes the error between the original velocity curve and the reconstructed one, using the delta-lognormal equation. Given that there is no guarantee that the process will always lead to a global optimum, there are instances where the algorithm might get trapped in a local inconsistent minimum with a negative *t*_0_.

### Statistical Analysis

As our main interest is to verify whether each group reacted to the 3-month AET, we first carried out simple repeated-measures ANOVA, separately for each group. The dependent variables were the kinematic parameters and the independent variable was the time of the assessment (pre- vs. post-AET). Whenever we observed significant changes in a single group, a repeated-measure ANCOVA model (the same dependent variables, but with group and time of assessment as independent variables) was used to test the effect of AET on primary and secondary outcomes in PD participants compared to HA subjects. Given that there were significant differences between the groups in terms of depression level and age at baseline, we used these variables as covariates in the model to statistically control for their effect when assessing group differences. The ANCOVA aimed to test for group differences across assessments (group ^∗^ assessment interaction), as well as the effect of training within each group after AET, for all kinematic movement parameters. In order to account for the effect of multiple comparisons, the statistical significance was adjusted using the Bonferroni method. Paired *t*-test was used to evaluate AET-related changes in UPDRS subscores in PD participants alone. In addition, the associations between exercise-related changes in upper-limb functioning, cardiovascular capacity, executive functions, and MSL in PD patients were tested using Pearson’s partial correlation (controlling for age and depression level). All results were expressed as means ± standard deviations for descriptive statistics. Analyses were conducted using SPSS 21.0 (IBM, Armonk, NY, United States: IBM Corp.). The level of statistical significance for all tests was set at *p* < 0.05.

## Results

Forty-four participants (21 PD patients and 23 HA) were eligible after the completion of the first evaluation. Two HA decided to withdraw from the project prior to commencing the AET regimen, for personal reasons. Two participants (1 HA and 1 PD) did not complete the program because of medical conditions external to the research project. One PD patient completed the AET, but was excluded from analysis because of unusually low levels of physical and cognitive performances (outlier: mean > 2 SD). Another PD patient was excluded from the analyses for technical reasons given that his drawing trials were not saved during one of the evaluations. A total of 38 persons (18 PD patients and 20 HA) were thus included in the final analysis. Demographic characteristics and initial values of the study participants are described in **Table 1**. The 3-month AET did not permit to observe any change in the UPDRS III in PD participants, whether in total, at the level of tremor, rigidity, or the right upper limb (**Table 2**).

**Table 1:** Demographic data.

Characteristics	HA	PD	Group differences
Age (years)	64 ± 8.19	59 @ 7.11	*p* = 0.06
Ratio men/women	8/12	13/6	*p* = 0.07
Education (years)	15.7 ± 2.36	15.05 @ 2.78	*p* = 0.43
Cognition (MMSE/MoCA)	29.18 ± 1.25	28.4 @ 1.34	*p* = 0.28
	29.56 ± 1.51	27.21 @ 1.85	*p* = 0.08
Depression (BDI)	4.8 ± 4.5	10.5 @ 8.3	*p* < 0.01
Anxiety (BAI)	2.1 ± 2.7	8.6 @ 9.4	*p* < 0.01
Inhibition (Stroop, in s)	115.4 ± 4.7	128.5 @ 6.7	*p* = 0.12
Flexibility (TMT, in s)	75.0 ± 6.4	85.5 @ 10.5	*p* = 0.39
UPDRS III	N/A	21.84 @ 6.16	N/A
Duration of disease (years)	N/A	8.1 @ 9.12	N/A
H&Y	N/A	2.1 @ 0.2	N/A

*Means ± SD. HA, healthy adults; PD, Parkinson’s diseases patients; N/A, non-applicable; s, seconds*.

**Table 2:** Kinetic parameters of the fast simple reaction time task and motor symptoms examination.

Kinematic variables	HA		PD		Statistical significance		
	Pre-AET	Post-AET	Pre-AET]	Post-AET	Interaction	AET	Group
SNR	25.6 @ 4.3	26.3 @ 4.7	24.1 @ 3.9	23.5 @ 2.7	0.151	0.503	0.116
Number of trials	23.4 @ 5.2	21.7 @ 7.2	17.8 @ 8.1	20.6 @ 5.3	0.119	0.606	0.442
*t*_0_	0.17 @ 0.08	0.17 @ 0.08	0.15 @ 0.07	0.16 @ 0.06	0.555	0.306	0.706
*D*_1_	119.4 @ 11.2	122.8 @ 9.9	126.6 @ 15.8	115.1 @ 7.3^†^	0.006^∗^	0.719	0.965
μ_1_	-1.17 @ 0.34	-1.19 @ 0.42	-0.86 @ 0.37	-0.95 @ 0.37	0.675	0.283	0.055
σ_1_	0.29 @ 0.06	0.27 @ 0.05	0.28 @ 0.05	0.28 @ 0.05	0.957	0.207	0.934
*D*_2_	22.1 @ 6.5	22.6 @ 6.9	30.8 @ 14.1	22.8 @ 5.0^†^	0.052	0.683	0.064
μ_2_	-0.81 @ 0.32	-0.84 @ 0.39	-0.57 @ 0.33	-0.64 @ 0.32	0.824	0.324	0.128
σ_2_	0.12 @ 0.02	0.11 @ 0.02	0.13 @ 0.03	0.11 @ 0.01^†^	0.027^∗^	0.236	0.226
ET	0.72 @ 0.15	0.70 @ 0.19	0.88 @ 0.23	0.83 @ 0.19	0.489	0.490	0.027^∗^
RT	0.33 @ 0.10	0.34 @ 0.13	0.37 @ 0.11	0.37 @ 0.10	0.487	0.064	0.512
MT	0.35 @ 0.10	0.33 @ 0.10	0.48 @ 0.17	0.42 @ 0.12^†^	0.133	0.671	0.007^∗^
UPDRS III
Tremor	N/A	N/A	1.08 @ 1.26	1.08 @ 1.56		1.000	
Rigidity	N/A	N/A	4.72 @ 2.65	3.81 @ 2.43		0.124	
Right UL	N/A	N/A	4.61 @ 1.53	4.58 @ 1.78		0.923	
Total	N/A	N/A	21.92 @ 6.32	21.53 @ 6.38		0.765	

*Means ± SD. ^†^A significant within-group difference from baseline. ^∗^A significant effect for interaction or mean effects. HA, healthy adults; PD, Parkinson’s disease patients; AET, aerobic exercise training; SNR, signal-to-noise ratio (in decibels); number of trials, number of successful trials; *t*_*0*_, time occurrence of the input commands to the neuromuscular system; *D*_*1*_, agonist component; μ_*1*_, time delay of *D*_*1*_; σ_*1*_, response spread of *D*_*1*_; *D*_*2*_, antagonist component; μ_*2*_, time delay of *D*_*2*_; σ_*2*_, response spread of *D*_2_; ET, time between visual stimulus and the immobilization of the stylus; RT, reaction time; MT, movement time; UL, upper limb, includes the following items: tremor at rest, postural tremor, rigidity of arm, finger taps, hand movements, rapid alternating movements of hands*.

We observed significant difference between PD and HA groups at baseline in regards to three variables: *D*_2_ (*p* < 0.05), ET (*p* < 0.05), and MT (*p* < 0.01), suggesting that PD individuals had a larger antagonist response, a longer ET between the visual stimulus and the end of the movement, and longer time of displacement of the stylus before the exercise training program began.

**FIGURE 2:**
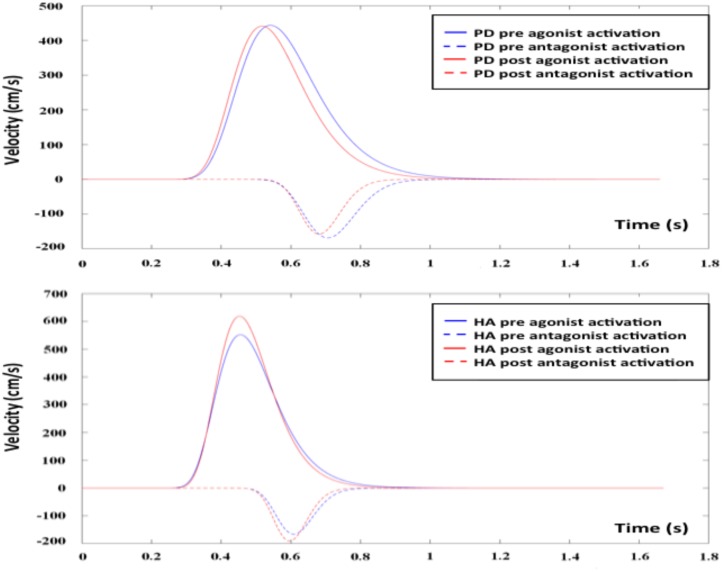
Agonist and antagonist responses before and after AET for PD and HA groups. AET, aerobic exercise training; PD, Parkinson’s disease patients; HA, healthy adults.

**FIGURE 3:**
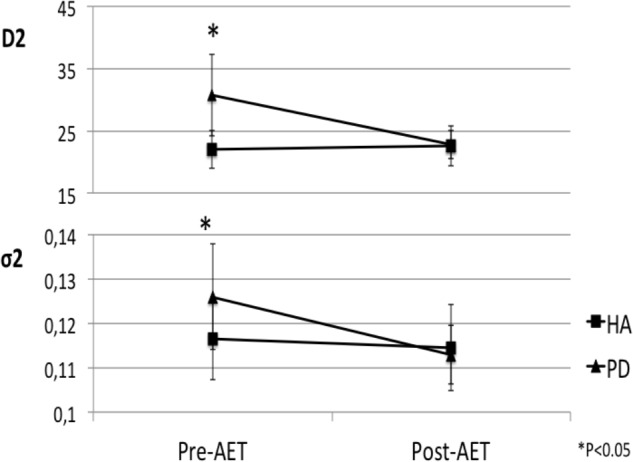
Normalization of antagonist parameters in PD group after AET. AET, aerobic exercise training; PD, Parkinson’s disease patients, HA, healthy adults; *D*_2_, antagonist component; σ_2_, response spread of *D*_2_.

Following the 12-week AET, the repeated measures ANOVA revealed that PD participants showed significant decreases of the *D*_1_ (*F*_1,17_ = 8.916, *p* < 0.01), *D*_2_ (*F*_1,17_ = 5.039, *p* < 0.05), and σ_2_ (*F*_1,17_ = 6.553, *p* < 0.05), the agonist command, the antagonist command, and its response spread, respectively. The mixed ANCOVA model revealed a significant group ^∗^ session interaction effect for *D*_1_ (*F*_1,34_ = 8.679, partial *R*^2^ = 0.203, *p* < 0.01) and σ_2_ (*F*_1,34_ = 5.359, partial *R*^2^ = 0.136, *p* < 0.05) (**Table 2**), suggesting a significant change for these two parameters only in PD patients following AET (**Figure 2**). While differences were observed at baseline for *D*_2_, ET, and MT, the groups did not differ significantly in post-AET comparisons for *D*_2_ and ET (**Figure 3**).

As reported previously by our research group (Duchesne et al., 2015, 2016), significant between-sessions differences were found in both groups for outcomes related to aerobic capacity (VO2 peak), MSL capacity, and cognitive inhibition (all *p* < 0.05), indicating that the training improved participants’ fitness, procedural learning, and cognitive inhibition, regardless of the health status (see Supplementary Material, Nadeau et al., 2017, AET-related changes in various domains). However, in the current study, we tested for correlations between these variables and kinematic parameters that showed significant AET-related changes (*D*_1_, *D*_2_, σ_2_, MT), among PD participants. We observed a significant association between the change in cognitive inhibition and σ_2_ (*r* = -0.560, *p* < 0.05, *df* = 14), as well as between AET-related change in inhibition and the change in MT (*r* = -0.531, *p* < 0.05, *df* = 14). These results indicate that an increase in response spread of the antagonist component or in MT is associated with a decrease in inhibition effect. No correlation was observed between any of the kinematic parameters and the patients’ aerobic or MSL capacities.

## Discussion

In the current study, we investigated the effects of an AET regimen using stationary bicycling on kinematic parameters of an arm movement in sedentary HA and in PD patients. As reported previously by our group (Duchesne et al., 2015, 2016), such training regimen improved cardiovascular capacity, executive functions, and motor learning capacities in both groups. Here, we report that AET also had a significant positive impact on the capacity to draw lines in a more efficiency way, in PD patients, indicating an improvement in the upper limb motor function. Specifically, after AET there was a significant decrease of the antagonist response of the movement (*D*_2_), an amelioration that can be interpreted as an improvement in the control of the motor command in PD patients. Other AET-related changes included a better response spread of the antagonist activation (σ_2_), which may reflect a more global modification of the CNS in improving its response time. The significant improvement in MT in the context of no change in the RT also demonstrates AET-related improvements in the capacity to execute a faster movement, while the time needed from the brain to process the information of the visual stimulus remained the same in PD patients. Although we did not observe any changes in motor symptoms (UPDRS III), this last result suggests that the improvements measured while drawing lines are not due to a decrease in rigidity, for example. By contrast to the changes observed in the PD group, no significant AET-related changes in the kinematic parameters were observed in the HA group.

Initial group differences for ET and *D*_2_ variables disappeared after AET, suggesting an effect of “normalization” due to improvements in PD patients. For the MT, even if there was a significant AET-related change in PD patients, the difference between the two groups was still present after AET. To our knowledge, this is the first time a “normalization” effect is reported following AET in PD patients in regards to the upper limb motor function.

We found a significant negative relationship between changes in cognitive inhibition of PD participants and their change in the MT or response spread of the antagonist activation (σ_2_), indicating that individuals who improved on one variable showed a decrease in the other after AET. Although conjectural, these results suggest that in this specific population, there may be a trade-off between improving motor function and executive function. It may also indicate the presence of two clinical sub-populations in our sample, a mixture of patients who are more impaired on either executive or motor functions hence responding differently to the intervention and making the correlation spurious. Further studies should explore this issue.

To date, studies on physical exercise training have proposed several action mechanisms underlying this type of intervention. The simplest one is that increase of heart rate and blood pressure during exercise could help to increase effectiveness of PD medication by making it more easily pass the blood–brain barrier (Speelman et al., 2011). Also, some studies have reported an increase in brain-derived neurotrophic factors (BDNFs) and glia cell line-derived neurotrophic factor (GDNF), neurotrophins known to regulate survival and activity of dopaminergic neurons, following short bouts of aerobic exercise in PD patients (Frazzitta et al., 2014; Zoladz et al., 2014; Marusiak et al., 2015). Moreover, Marusiak et al. (2015) reported that this increase in BDNF levels correlated with improvements in PD rigidity. Another proposed action mechanism has been related to neurotransmitters, as that progressive aerobic exercise could lead to an increase in dopamine *D*_2_ receptor density within the regional boundaries of the dorsal striatum [observed with PET imaging (Fisher et al., 2013)]. Similarly, there is evidence for an increase in corticomotor excitability (observed via transcranial magnetic stimulation) in Parkinsonian individuals (Fisher et al., 2008). It is important to highlight the fact that our participants used primarily the lower limbs during AET; yet we observed changes in the upper limb. This would suggest that functional changes in corticospinal pathways may occur at multiple levels, not only at those directly involved in generating and controlling the limbs performing the movements. In support of such mechanisms are the findings of a work performed by Zhou et al. (2017) that demonstrate changes in the motor-evoked potentials in the lower limb in neurologically intact individuals and patients with incomplete spinal cord injury following arm cycling alone or simultaneous arm and leg cycling, respectively. Our study adds to this body of knowledge, by providing evidence that AET can improve the synergistic action of an agonist and antagonist neuromuscular networks in PD. Whether this improvement in motor control is achieved via one or some of these neurophysiological mechanisms remains to be explored in future research.

A third possible mechanism of action could be that AET may lead directly to structural and/or functional changes in the brain. Indeed, many neuroimaging studies have already reported differences in gray and white matter between HA and PD subjects (Lehéricy et al., 2012; Meijer et al., 2013; Agosta et al., 2016; Al-Radaideh and Rababah, 2016), as well as in functional activity at rest or during various tasks (Mallol et al., 2007; Sharman et al., 2012; Caproni et al., 2013; Skidmore et al., 2013; Nigro et al., 2016). However, to date, only one study reported the effect of exercise training on the neural correlates of MSL in PD (Duchesne et al., 2016).

Contrary to other studies using FE to improve upper limb, we chose to use VE instead. FE is described as an aerobic exercise in which the rate is augmented mechanically to assist the participant, hence allowing the achievement and maintenance of an exercise rate greater than the preferred voluntary rate of exercise (VE). Consequently, it has been hypothesized that the magnitude of intrinsic feedback provided in FE could permit the release of a greater amount of dopamine than VE, which could then have a greater positive impact on the brain structure and function in PD (Alberts et al., 2011). In fact, it has been proposed that lower-extremity FE could produce global improvements in motor symptoms using the same pathways through which anti-PD medication acts to produce symptomatic relief in individuals with PD (Alberts et al., 2016). For this reason, it has also been suggested that FE could be a better way to exercise for people affected by PD. However, from a clinical and practical perspective, FE devices are not easily accessible to the general public, including people suffering from PD. Furthermore, given that our study shows that there are significant beneficial effects when using VE, we thus believe that this latter type of training is more feasible in clinical settings.

The fact that we observed improvements in kinematic parameters, but not in the UPDRS scores, after AET suggests that our task using the delta-lognormal model (Plamondon et al., 1993, 2003; Plamondon, 1995a,b, 1998) may be a more sensitive mean to assess changes in motor function (and indirectly, motor symptoms) following treatment in PD. Even though accelerometers and gyroscopes could be used to record three-dimensional motions and to quantify more objectively tremor and bradykinesia during the different tasks composing the motor examination of the UPDRS (Heldman et al., 2014), such a setup does not offer any insight into the neurophysiological mechanisms underlying the motor symptoms (i.e., agonist and antagonist muscle activity during motor task performance). The original contribution of the current study is thus the use of a relatively simple kinematic task, which can be easily performed by PD patients and, most importantly, does offer an indirect and objective clinical measure of the state of the global neurophysiological mechanisms involved in controlling the upper limb. Indeed, it must be remembered that the lognormal impulse response predicted by the kinematic theory is the optimal function describing the neuromuscular system of human subjects in perfect control of their movements (Djioua and Plamondon, 2010). As a person get old, he/she will depart from this ideal behavior more or less severely, depending on his/her health status (Plamondon et al., 2013b). In this context, the SNR can be seen as an objective parameter that characterizes the global motor behavior of a subject. The higher it is the better is the motor control. Moreover, the delta-lognormal model proposes a complementary and new window to analyze and interpret a movement kinematics in terms of agonist (*D*_1_) and antagonist (*D*_2_) input commands, which reflects the intention of a subject, as *D*_1_-*D*_2_ is equal to the physical distance covered by a given movement. Similarly, the occurrence of these commands at *t*_0_ has been shown to be directly correlated to a specific ERP potential occurring at *t*_0_ (O’Reilly et al., 2013). Similarly, the timing parameters μ and σ indirectly reflect the muscle coupling through the proportionality of their cumulative time delays, as observed via EMG data (Plamondon et al., 2013a). In other words, reconstructing each velocity profile with the delta lognormal model, a neuroscientist get access to physiologically meaningful global parameters describing the status of the agonist and antagonist neuromuscular system of a subject, can monitor its time evolution and estimate if he or she is improving, stays stable, or deteriorates.

One limitation of the current study was the lack of a PD control group for the type and intensity of exercise (e.g., a PD group undertaking another type of training regimen). Also, having more than the two pre- and post-AET assessments would have allowed the mapping of the trajectory of changes during training. Another limitation stems from the use of a mathematical model (kinematic theory) to infer the neurophysiological changes in the motor system; even though this model has been found to have some physiological plausibility, supported previously with EEG and EMG, it remains nevertheless an indirect assessment of these mechanisms. EEG or EMG would have to be used in future research to corroborate the findings of the present study. Despite these constraints, however, our findings indicate that using VE and a typical stationary bicycle can still lead to great improvements in the upper limb movement fluidity, in addition to aerobic capacity, executive function, MSL, and in gait. Finally, we believe that our study results contribute to the field and may inspire future research about how exercise could help to improve activities of daily living relying on the motor function of the upper limb in people with PD.

## Author Contributions

JD had full access to all of the data in the study and took responsibility for the integrity of the data and the accuracy of the data analysis. He was responsible for design, funding, and conduct of the study. AN and CD managed the study. AN, OL, and RP led the statistical analyses. AN wrote the manuscript. AN, OL, RP, AB, and JD were involved in data interpretation. OL, RP, AB, CD, M-ÈR, FB, FG, A-LL, LB, and JD reviewed and approved the manuscript.

## Conflict of Interest Statement

The authors declare that the research was conducted in the absence of any commercial or financial relationships that could be construed as a potential conflict of interest.
